# A Question-and-Answer System to Extract Data From Free-Text Oncological Pathology Reports (CancerBERT Network): Development Study

**DOI:** 10.2196/27210

**Published:** 2022-03-23

**Authors:** Joseph Ross Mitchell, Phillip Szepietowski, Rachel Howard, Phillip Reisman, Jennie D Jones, Patricia Lewis, Brooke L Fridley, Dana E Rollison

**Affiliations:** 1 Department of Machine Learning H Lee Moffitt Cancer Center and Research Institute Tampa, FL United States; 2 Department of Medicine, Faculty of Medicine & Dentistry, and the Alberta Machine Intelligence Institute University of Alberta Edmonton, AB Canada; 3 Alberta Health Services Edmonton, AB Canada; 4 Department of Health Data Services H Lee Moffitt Cancer Center and Research Institute Tampa, FL United States; 5 Department of Biostatistics and Bioinformatics H Lee Moffitt Cancer Center and Research Institute Tampa, FL United States

**Keywords:** natural language processing, NLP, BERT, transformer, pathology, ICD-O-3, deep learning, cancer

## Abstract

**Background:**

Information in pathology reports is critical for cancer care. Natural language processing (NLP) systems used to extract information from pathology reports are often narrow in scope or require extensive tuning. Consequently, there is growing interest in automated deep learning approaches. A powerful new NLP algorithm, bidirectional encoder representations from transformers (BERT), was published in late 2018. BERT set new performance standards on tasks as diverse as question answering, named entity recognition, speech recognition, and more.

**Objective:**

The aim of this study is to develop a BERT-based system to automatically extract detailed tumor site and histology information from free-text oncological pathology reports.

**Methods:**

We pursued three specific aims: extract accurate tumor site and histology descriptions from free-text pathology reports, accommodate the diverse terminology used to indicate the same pathology, and provide accurate standardized tumor site and histology codes for use by downstream applications. We first trained a base language model to comprehend the technical language in pathology reports. This involved unsupervised learning on a training corpus of 275,605 electronic pathology reports from 164,531 unique patients that included 121 million words. Next, we trained a question-and-answer (Q&A) model that connects a Q&A layer to the base pathology language model to answer pathology questions. Our Q&A system was designed to search for the answers to two predefined questions in each pathology report: *What organ contains the tumor?* and *What is the kind of tumor or carcinoma?* This involved supervised training on 8197 pathology reports, each with ground truth answers to these 2 questions determined by certified tumor registrars. The data set included 214 tumor sites and 193 histologies. The tumor site and histology phrases extracted by the Q&A model were used to predict International Classification of Diseases for Oncology, Third Edition (ICD-O-3), site and histology codes. This involved fine-tuning two additional BERT models: one to predict site codes and another to predict histology codes. Our final system includes a network of 3 BERT-based models. We call this CancerBERT network (caBERTnet). We evaluated caBERTnet using a sequestered test data set of 2050 pathology reports with ground truth answers determined by certified tumor registrars.

**Results:**

caBERTnet’s accuracies for predicting group-level site and histology codes were 93.53% (1895/2026) and 97.6% (1993/2042), respectively. The top 5 accuracies for predicting fine-grained ICD-O-3 site and histology codes with 5 or more samples each in the training data set were 92.95% (1794/1930) and 96.01% (1853/1930), respectively.

**Conclusions:**

We have developed an NLP system that outperforms existing algorithms at predicting ICD-O-3 codes across an extensive range of tumor sites and histologies. Our new system could help reduce treatment delays, increase enrollment in clinical trials of new therapies, and improve patient outcomes.

## Introduction

### Background

Much of the information in electronic medical records (EMRs) required for the practice of clinical oncology and cancer research is contained in unstructured text. As much as 80% of EMR data can be found in narrative text notes and scanned documents [1], ranging from clinic or surgical notes, including pathology, radiology, and ambulatory care, to past medical or family history. The extraction of discrete data elements from this unstructured text, particularly those relating to disease diagnosis and commonly captured in routine pathology reports, is critical for the selection of treatment options, identification of patients eligible for clinical trials, and monitoring of adherence to established clinical treatment pathways.

Natural language processing (NLP) has been used to extract information from medical text for several decades [2-6], and a thorough review of NLP-based information extraction for cancer-related EMR notes can be found in the study by Datta et al [7]. Application of NLP to pathology reports has also been prevalent in the literature during the course of the last decade [8-19]. That said, many early studies used regular expression– and rule-based systems [20,21] that require considerable up-front development and can be difficult to adapt and maintain.

More recently, there has been growing interest in more highly automated deep learning approaches for clinical NLP [6,22]. In late 2018, a powerful new deep learning NLP algorithm was released: bidirectional encoder representations from transformers (BERT) [23]. BERT established new, state-of-the-art performance levels on common nonclinical NLP benchmarks [24]. This success spawned rapid research and development of multiple BERT-inspired and transformer-based neural architectures [25-33]. Several of these have, for the first time, achieved or surpassed human-level performance on tasks as diverse as question answering, named entity recognition, speech recognition, and more [30,33-35]. BERT and related architectures have facilitated significant improvements in multiple medical applications, including processing of electronic health records [36,37], outcome prediction [38-40], identification of medical terms and concepts [41], medical chatbots [42], sentiment analysis [43], recommender systems [44], and others. Despite BERT’s success, we are aware of only a single application of BERT to the already promising area of free-text pathology reports [45]. The study focused on classification of text into only a few cancer-related categories, including afflicted organ (15 organ groups), disease type (noncancer, premalignant, or cancer), cancer reason (6 histology groups), and presence of metastatic disease (no, yes: in lymph nodes and yes: in non–lymph node tissue). Our goal is to develop and evaluate a BERT-based system to extract detailed tumor site and histology information from free-text pathology reports. The availability of manually curated data within the H Lee Moffitt Cancer Center and Research Institute (Moffitt) Cancer Registry (MCR) represented a unique opportunity to train a BERT-based system using a gold standard data set classified using a standard ontology.

BERT’s proficiency at question answering prompted us to construct a question-and-answer (Q&A) system to extract clinical data from pathology reports. This concept has long been compelling—Q&A systems for medical data extraction have been pursued for >40 years [46]. Such a system would have several desirable properties: an intuitive user interface, the ability to extract additional data fields by searching for answers to additional questions, and the ability to generalize to other medical documents. Furthermore, it would allow us to make data available for clinical and research use close to real time, thus reducing treatment delays, increasing enrollment in clinical trials of new therapies, and improving patient outcomes. To train such a general-purpose Q&A system on pathology reports, one would need a diverse set of questions on which to train it. Our task in this paper is more modest (and is in essence a classification task of site and histology); however, we view the Q&A portion of our system as a small step toward this broader goal.

### Primary Contributions

This work describes three primary contributions:

1. A new BERT language model for comprehension of pathology reports in oncology. We call this *CancerBERT*, or *caBERT* for short.
2. A new Q&A caBERT-based system, tolerant to varied terminologies, word orders, and spelling mistakes, to extract tumor site and histology descriptions from free-text pathology reports.
3. A new caBERT network (caBERTnet) to predict International Classification of Diseases for Oncology, version 3.2 (ICD-O-3.2), codes from the extracted descriptions. This system can handle up to 332 organ sites and 1143 tumor histologies. On an unseen test data set with 214 sites and 193 histologies it achieved overall accuracies that are equal to, or above, those of existing systems, while also expanding on the number of site and histology classes captured by these systems. Although the results in practice still require human validation, they provide a means of early abstraction from unstructured pathology text over a very broad set of sites and histologies and in addition can provide an initial signal to assist expert cancer registrars in their case diagnosis–abstraction workflow.

## Methods

Information on our software development tools is provided in Multimedia Appendix 1 [47-53]. To construct our system, we had to achieve three specific aims: (1) extract accurate tumor site and histology descriptions from complex free-text pathology reports, (2) accommodate the diverse terminology used to indicate the same pathology, and (3) provide accurate standardized tumor site and histology codes for use by downstream applications.

### Extract Tumor Site and Histology Descriptions

#### Overview

Constructing this system required us to first train a base language model to comprehend the technical language in pathology reports (Textbox 1). This involved *unsupervised* training on a large corpus of pathology text. For this we constructed a *pathology language–model training data set*, described in more detail in the next section (*Pathology Language Model*).

**A fragment of text from a pathology report generated at H Lee Moffitt Cancer Center and Research Institute (Moffitt).**

**Fragment of text from a pathology report generated at Moffitt**

- clinical history: not given. preoperative diagnosis: right lower lobe, squamous cell carcinoma. specimen types: a: right station 7 fs b: station # 10 c: right lung fs d: station 4r e: additional station 10 f: additional station 7 final diagnosis: a. lymph node right station 7 biopsy: anthracotic lymph node 1 with lymphoid hyperplasia negative for malignancy. b. lymph node station 10 biopsy: anthracotic lymph node 1 negative for malignancy. c. lung right pneumonectomy: moderate to poorly differentiated squamous cell carcinoma with extensive necrosis see key pathological findings. bronchial and vascular resection margins negative for malignancy. three of 10 hilar lymph nodes with metastatic squamous cell carcinoma. d. lymph nodes station 4r biopsies: anthracotic lymph nodes 5 negative for malignancy. e. lymph node additional station 10 biopsy: minute lymph node 1 negative for malignancy. f. lymph node station 7 biopsy: anthracotic lymph nodes 4 negative for malignancy. key pathological findings tumor type: squamous cell carcinoma with extensive necrosis. histological grade: moderate to poorly differentiated. tumor location: right lung involving right lower lobe. right upper and middle lobes free of tumor.

Next, it required us to train a Q&A model that appends a Q&A layer onto the pathology language model to answer pathology questions. Our Q&A system was designed to search for the answers to two predefined questions in each pathology report:

1. What organ contains the tumor?
2. What is the kind of tumor or carcinoma?

For example, when presented with the report shown in Textbox 1, the system should respond to the question *What organ contains the tumor?* with *C343: lower lobe, lung*, and would respond to the question *What is the kind of tumor or carcinoma?* with *8070/3: squamous cell carcinoma, nos* (not otherwise specified).

This involved *supervised* training on a set of pathology reports, each with ground truth answers to these 2 questions determined by human experts. To do this, we constructed a second *fine-tuning training data set*, described in more detail in the *Pathology Q&A Model* section. Finally, we evaluated our system using a sequestered *fine-tuning testing data set*, described in more detail in the *Pathology Q&A Model* section.

#### Pathology Language Model

**Figure 1:**
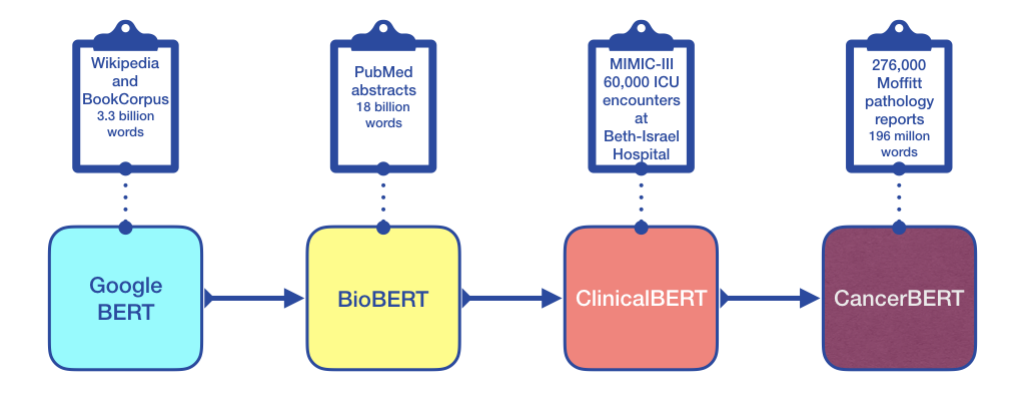
Sequence of transfer-learning steps used in training the CancerBERT base language model. BERT: bidirectional encoder representations from transformers; ICU: intensive care unit; MIMIC-III: Medical Information Mart for Intensive Care, version 3.

Training a base language model to comprehend pathology reports leveraged prior work by several groups (Figure 1). Lee et al [54] performed transfer learning on BERT using nearly 18 billion words extracted from PubMed abstracts. The result, BioBERT, is tuned for biomedical language comprehension tasks and is publicly available. Next, Alsentzer et al [55] performed transfer learning on BioBERT to tune it for clinical language comprehension. They used EMR notes in the Medical Information Mart for Intensive Care, version 3 (MIMIC-III) data set [56], which includes data from approximately 60,000 intensive care unit stays by patients at Beth-Israel Hospital in Boston, Massachusetts. The model created by Alsentzer et al [55], ClinicalBERT, was also made publicly available.

Alsentzer et al [55] created two models built upon BioBERT: one trained on all MIMIC-III notes and one trained on just the MIMIC-III discharge summaries. Initial pretraining experimentation revealed that the latter provided higher accuracies on a separate sample of our pathology reports. We noted that Moffitt pathology reports have a language structure closer to discharge summaries than to general clinical notes. Consequently, our model was initialized with weights from the latter of the two ClinicalBERT models: ClinicalBERT–Bio+Discharge Summary BERT Model.

Transfer learning was accomplished by performing masked-language modeling [23]. Briefly, 15% of the words in the corpus are selected at random and replaced with a *mask* token. The language model is then trained to predict the masked words. The word-masking process is performed automatically at the beginning of each training run.

Our language-model training corpus included electronic pathology reports of solid tumors produced by pathologists at Moffitt between 1986 and 2020. The year 1986 was the earliest date on pathology reports cataloged in our enterprise data warehouse. The data set was restricted to solid tumors for two reasons: first, to focus the problem domain for this proof-of-concept study, and, second, Moffitt hematologic pathology reports follow a quasi-structured format, reducing the need for extraction of data from unstructured text.

This data set contained both Health Level Seven International messages and plain-text pathology reports. These were minimally processed to extract and clean the text relevant to pathology (more details can be found in Multimedia Appendix 1). The final language model training corpus included 275,605 electronic pathology reports from 164,531 unique patients and included 121 million words.

#### Pathology Q&A Model

**Figure 2:**
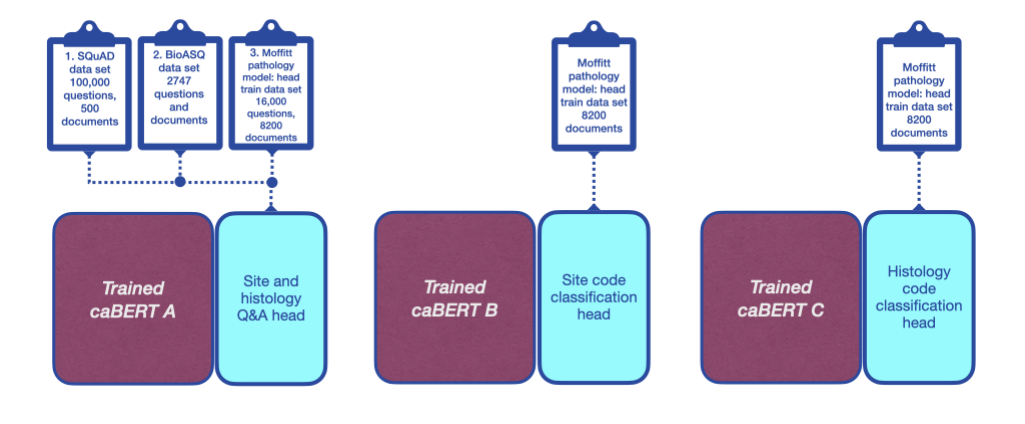
Lesson plan for the caBERT network consisting of one question and answering model A and two classification models, one for primary site (model B) and another for histology (model C). BioASQ: Biomedical Semantic Indexing and Question Answering; Q&A: question and answer; SQuAD: Stanford Question Answering Dataset.

The pathology Q&A lesson plan involved 3 stages, each intended to improve our system’s comprehension of pathology reports and thereby increase the accuracy of question answering (Figure 2). The three stages involved training the Q&A model to (1) answer general English language questions, (2) answer technical biomedical science questions, and (3) answer questions from Moffitt pathology reports.

Each training stage used supervised learning. This required a training data set that included passages of text, ≥1 questions related to each passage, and ground truth answers to those questions that appeared as contiguous phrases within the related passage. At the end of each stage we evaluated our system using the same sequestered test data set constructed from Moffitt pathology reports, as described in more detail later in this section. The experimental parameters used to train the Q&A model were held constant over all stages and are listed in Table S1 in Multimedia Appendix 1.

For the first stage of training we used the Stanford Question Answering Dataset (SQuAD), version 1.1 [57]. SQuAD consists of more than 100,000 questions and answers created by crowdworkers on Wikipedia articles. The SQuAD data format is widely used in NLP research. Therefore, we designed our system to read and process data sets in this format.

For the second stage of training we used the large-scale Biomedical Semantic Indexing and Question Answering (BioASQ) data set [58]. In particular, we used data from BioASQ Challenge 7b: Biomedical Semantic Question Answering. This data set contains 2747 training questions along with their ground truth answers. According to the BioASQ Challenge 7b description: “All the questions are constructed by biomedical experts from around Europe.” This data set was converted to SQuAD format by Yoon et al [59] and made available for public use.

Next, we constructed a Q&A fine-tuning data set in SQuAD format based on Moffitt pathology reports. We obtained ground truth answers to our 2 questions from data abstracted by Moffitt certified tumor registrars (CTRs). CTRs undergo an extensive training and internship program to become proficient at extracting quantitative and categorical data from unstructured pathology reports. They are widely employed by cancer centers and other organizations to extract data for clinical and research applications and for reporting to state and national agencies. The MCR deploys state-of-the art quality assurance procedures: its benchmark for quality is 90% and its target accuracy is 95% [60].

**Figure 3:**
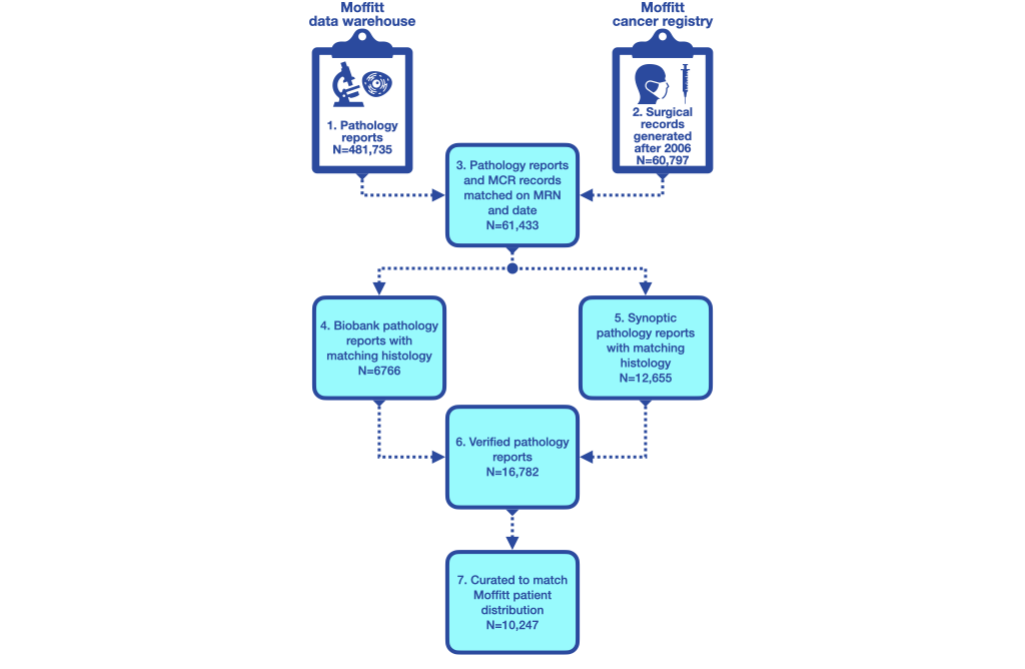
Flowchart depicting the data curation process for creating the Moffitt fine-tuning data sets (used in the site and histology question-and-answer and classification tasks). MCR: Moffitt Cancer Registry; MRN: medical record number.

Moffitt’s enterprise data warehouse was searched to find solid tumor pathology reports generated after 2006 with matched MCR data (Figure 3). These reports were screened to ensure that they contained a description of a positive diagnosis of a single primary tumor. Next, each report was processed to ensure that it contained an answer to at least one of the questions in the Q&A model. This was accomplished programmatically by searching each report for a phrase contained in a table of acceptable answer phrases (Figure S1 in Multimedia Appendix 1), as described in more detail in the next section (*Accommodate Diverse Terminology*). Our search produced 16782 reports that met these inclusion criteria (Figure 3).

Next, we curated these reports to ensure that (1) the relative frequencies of the 10 most common tumor sites and histologies in this collection matched the relative frequencies in Moffitt’s patient population as a whole and (2) all MCR-assigned tumor sites and histologies reported for Moffitt patients after 2006 were represented in the data set. The final curated collection contained 10,247 reports (Figure 3). We will refer to this data set as the *fine-tuning* data set to contrast it with the caBERT language model data set used to train the base language model as described in the *Pathology Language Model* section.

**Figure 4:**
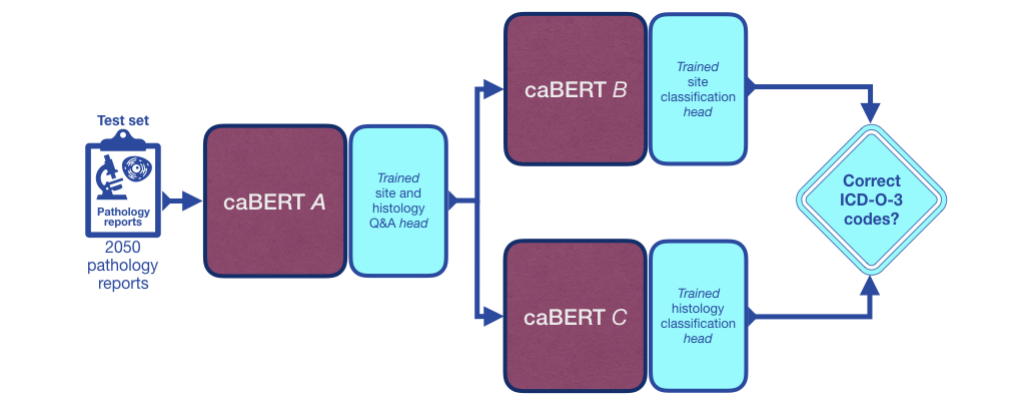
The final caBERT network (caBERTnet) connects caBERT instances A, B, and C, used for site and histology question and answering, International Classification of Diseases for Oncology, Third Edition (ICD-O-3) primary site and ICD-O-3 histology code classification, respectively.

The curated collection of reports was randomly divided to create two data sets: 79.99% (8197/10,247) of the reports were used to create the Moffitt fine-tuning training data set and 20.01% (2050/10,247) were used to create the Moffitt fine-tuning testing data set. Each data set was saved in SQuAD format using a custom-written Python program. The training data set was used for the final stage of Q&A training and also for ICD-O-3 code predictions, described in more detail in the *Accurate ICD-O-3 Codes* section. The testing subset was used to evaluate the impact of Q&A training at the end of each training stage (Figure S2 in Multimedia Appendix 1) and also to evaluate the performance of the final pipeline (Figure 4).

Question-answering accuracy was evaluated using two metrics: exact match and F1 score. Exact match is true if the caBERT-extracted phrase is an identical word-for-word match with the MCR phrase and false otherwise. We calculated the average number of true results across all test samples. The F1 score is a measure of the degree of overlap among the words in the caBERT-extracted phrase and the MCR phrase. This varies from 0 (no words in common) to 1 (all words in common but not necessarily in the same order). Each exact match corresponded to an F1 score of 1.0. We calculated the average F1 score across all test samples and expressed it as a percentage.

After all training stages were complete, they were repeated using the initial ClinicalBERT model (*ClinicalBERT–Bio+Discharge Summary BERT*) with a new randomly initialized Q&A layer. This allowed us to determine the impact of developing a pathology-tuned BERT model on extraction accuracy over the baseline accuracy of using ClinicalBERT alone (Figure S2 in Multimedia Appendix 1). The training parameters were set to those optimized for ClinicalBERT and reported by Alsentzer et al [55].

### Accommodate Diverse Terminology

Our pathology reports came supplied with ground truth labels for primary site and histology in the form of ICD-O-3 codes [61], which were abstracted by Moffitt CTRs. To train the Q&A model we needed a method to determine the precise location within each pathology report of the actual text corresponding to these labels. This proved nontrivial owing to the rather diverse terminology within each pathology report used to refer to each primary site and histology.

To address this issue, we used data from several canonical sources. Our primary source was the ICD-O-3 standards [62], which we used to define the primary *preferred* terminology for each code. Within the ICD-O-3 standards there are 332 unique site codes and 1143 unique histology codes, each with accompanying preferred terms. Along with the preferred term, many codes also have an additional set of synonyms, which we stored together with the preferred term in a table of acceptable phrases for each code. In addition to the ICD-O-3 tables, we also used terminology from the National Cancer Institute’s Surveillance, Epidemiology, and End Results (SEER) program Site/Histology Validation List [62], along with the SEER Site-specific training module website [63].

To provide a little more detail, the specific sources we used to construct our acceptable phrase tables were as follows. For histology, we used the ICD-O-3.2 morphology table (version 15112019) [64] and supplemented this with terms from the SEER Site/Histology Validation List (version 20150918), current versions of which are both available in Microsoft Excel format from their respective websites. For the site terms, we used the ICD-O-3 mapping table maintained by the National Cancer Institute [65], supplemented again by the SEER Site/Histology Validation List. In addition, for the site terms we also scraped the tables contained in the SEER Site-specific learning module website for any new terms.

The aforementioned sources have the benefit of being subject to an international standard and are useful in designating preferred terms for each histology and site code. However, we should note that there do exist slight discrepancies between the World Health Organization–maintained ICD-O-3 coding standards and the North American Association of Central Cancer Registries coding guidelines, which are followed in the SEER materials. For simplicity, we chose to base our model on the ICD-O-3 standards, but this caveat may prove relevant for any future cancer registry applications of the model.

Although these sources provided us with preferred and alternative terminologies, they did not encompass the full range of language used for every label in our pathology reports, which often included things such as permutations of word orderings as well as acronyms and other typographical differences with the canonical terms. Of note, Moffitt CTRs routinely record a short description of the histology and site for every labeled pathology report in a text-based field. For each histology and site code, we appended these additional phrases to the list of synonyms of the preferred canonical terminology.

Using the sources described earlier, we created two hierarchical tree structures as illustrated in Table S2 in Multimedia Appendix 1: one to hold histology terms and one to hold site terms. To construct these trees, the histology and site codes were first grouped into broad morphology and site groups as specified in the ICD-O-3 tables. Within each group are a collection of specific codes, where each code has an associated preferred term, and a list of synonyms. For efficient searching, these trees were stored as JSON objects that were imported into Python as nested dictionaries and lists. See Figure S1 in Multimedia Appendix 1 for an example of an entry in our acceptable phrase table.

To search each pathology report for appropriate spans of text, we used the trees to construct a dictionary with keys provided by the specific site and histology codes and values provided by the associated acceptable phrase table. Using this dictionary, for each pathology report we implemented a simple search for an exact match from the list of preferred terms and synonyms for the labeled ground truth histology and site code, giving preference for the preferred term, followed by each synonym ordered by length (with the longest matching synonym given preference over the others).

Even with the diverse terminology within the acceptable phrase table for each code, not every pathology report contained an exact match within the list of allowed terms. For pathology reports that did not contain an exact match, we further refined the search by allowing for matches that only overlapped with a subset of the word tokens within each phrase, again giving preference to the longest synonyms and also using a set of stop terms to avoid overly general terminology. To capture potential word-ordering differences, we allowed these word token subsets to be constructed in an arbitrarily permuted order, which was made efficient by using the *itertools* module available as part of the Python Standard Library.

Using the aforementioned procedure, of the 10,247 pathology reports in the Moffitt fine-tuning training and testing data sets, we were able to find appropriate textual answers within 10,096 (98.53%) reports for primary site (with n=8070, 79.93%, in the training set and n=2026, 20.07%, in the testing set) and within 10,218 (99.72%) reports for histology (with n=8176, 80.02%, in the training set and n=2042, 19.98%, in the testing set).

### Provide Accurate ICD-O-3 Codes

#### Overview

The tumor site and histology phrases extracted by the Q&A model (Figure 2) were used to predict ICD-O-3 site and histology codes. This involved fine-tuning two additional copies of caBERT: one to predict site codes and the second to predict histology codes. Our final system (Figure 4) includes a network of 3 caBERT-based models. We refer to this system as caBERTnet.

#### Training the ICD-O-3 Site and Histology Code Classifiers

Classifier training parameters are described in more detail in Table S1 in Multimedia Appendix 1. Briefly, each caBERT instance was trained to perform a classification task: given an input phrase, predict the corresponding ICD-O-3 code. Classification tasks were trained using the Moffitt fine-tuning training data set (*Extract Tumor Site and Histology Descriptions* section). Training samples were screened to ensure that each contained ground truth site and histology codes and at least one site or histology phrase provided by the MCR. Missing site and histology phrases were filled using SEER preferred terms. These were identified by performing a lookup into the ICD-O-3 table using the site or histology code in the training sample.

After screening, the ground truth phrases were labeled and concatenated to form a single combined phrase. For example, if the MCR phrases were *lung lower lobe* and *squamous cell carcinoma,* then the combined phrase would be *site: lung lower lobe. histology: squamous cell carcinoma*. The combined phrase was used to train both the caBERT site classification model and the caBERT histology classification model. The use of a combined phrase allowed caBERTnet to leverage any correlation between site and histology to improve its performance. For example, astrocytomas are brain tumors. When caBERTnet encountered a previously unseen pathology report during the test phase with the combined phrase *site: frontal. histology: anaplastic astrocytoma*, it correctly predicted a brain site of *C711, frontal lobe* and a histology of *9401/3 astrocytoma anaplastic NOS* (not otherwise specified).

#### Testing the ICD-O-3 Site and Histology Code Classifiers

After training of the site and histology ICD-O-3 code classification models was complete, caBERTnet performance was evaluated using the sequestered Moffitt fine-tuning test data set described in the *Extract Tumor Site and Histology Descriptions* section. For each test sample, the MCR-generated site and histology phrases were used to create a *ground truth* combined phrase. Next, the site and histology phrases extracted by the Q&A stage of caBERTnet were used to create a *predicted* combined phrase. The predicted phrase was tokenized to prepare it for input into each classification model. Ground truth site and histology codes from the MCR were enumerated, as described earlier, and stored as true labels. Subsequently, the trained site and histology classification models were used to classify the tokenized *predicted* combined phrases for each test sample. The outputs from this classification, logits, were converted into probabilities, sorted, and converted back into ICD-O-3 codes as described earlier, labeled as *predicted* codes, and saved for further performance analysis.

caBERTnet performance was evaluated in 3 different ways. First, the top 5 accuracies were determined. This metric (or its inverse, the top 5 error rate) is commonly used to evaluate classification algorithms [66]. Briefly, it calculates the average probability that the correct site or histology code occurs within the top N predicted codes because N is varied from 1 through 5. Top 1 accuracy, the accuracy of the code scored most highly by the classification algorithm, is equivalent to precision, recall, and F1 score for this classification task.

Second, we examined the effect of culling or removing infrequently occurring codes. Our hypothesis was that the caBERT site and histology code classifiers suffer when they do not have enough training data to learn from. Therefore, to examine the effect of training sample size, we iteratively eliminated site and histology codes from the full 2050-sample test data set when the number of examples with a particular code in the training set (alone) fell below a specified threshold. We varied that threshold from 0 samples (no culling) to 35 samples in increments of 5 samples. At each culling threshold we recalculated the top 5 performance of the site and histology classifiers.

Third, we calculated the overall accuracy of predicting the correct *group* code for each site and histology code. *Group* codes occur higher up in the ontological tree and, as the name implies, encompass a group or range of related tumor sites or histologies. For example, the site codes *C341 upper lobe, lung* and *C349 lung, NOS* have the same group code: *C34 bronchus and lung*. The histology codes *8070/3 squamous cell carcinoma, NOS* and *8051/0 verrucous carcinoma* both have the same group code: *805-808 squamous cell neoplasms*. The ICD-O-3 ontology includes 82 group-level site codes covering the 332 fine-grained site codes. It includes 49 group-level histology codes covering the 1143 fine-grained histology codes.

Group codes are useful for search and summary applications. The group codes for both the predicted and ground truth fine-grained codes were determined by searching in the tree data structures described in the *Accommodate Diverse Terminology* section. For each fine-grained code, the search started at that code’s location in the tree and proceeded upward. Finally, we calculated the overall accuracy of prediction within each group code for both site and histology predictions.

## Results

### Model Accuracy

The accuracy of the both the ClinicalBERT and caBERT Q&A models when tested on the Moffitt fine-tuning testing data set improved at each Q&A training stage (SQuAD, BioASQ, and Moffitt training data; Figure S2 in Multimedia Appendix 1). ClinicalBERT had higher accuracy than caBERT on the Moffitt test set after each of the first 2 training stages. This suggests that the specialized pathology-language tuning reduced caBERT’s ability to learn from the SQuAD and BioASQ training data sets. However, caBERT outperformed ClinicalBERT after training on Moffitt pathology reports. This was true both for exact match (3254/4068, 79.99%, for caBERT vs 3069/4068, 75.44%, for ClinicalBERT) and F1 score (87.76% for caBERT vs 84.85% for ClinicalBERT).

**Figure 5:**
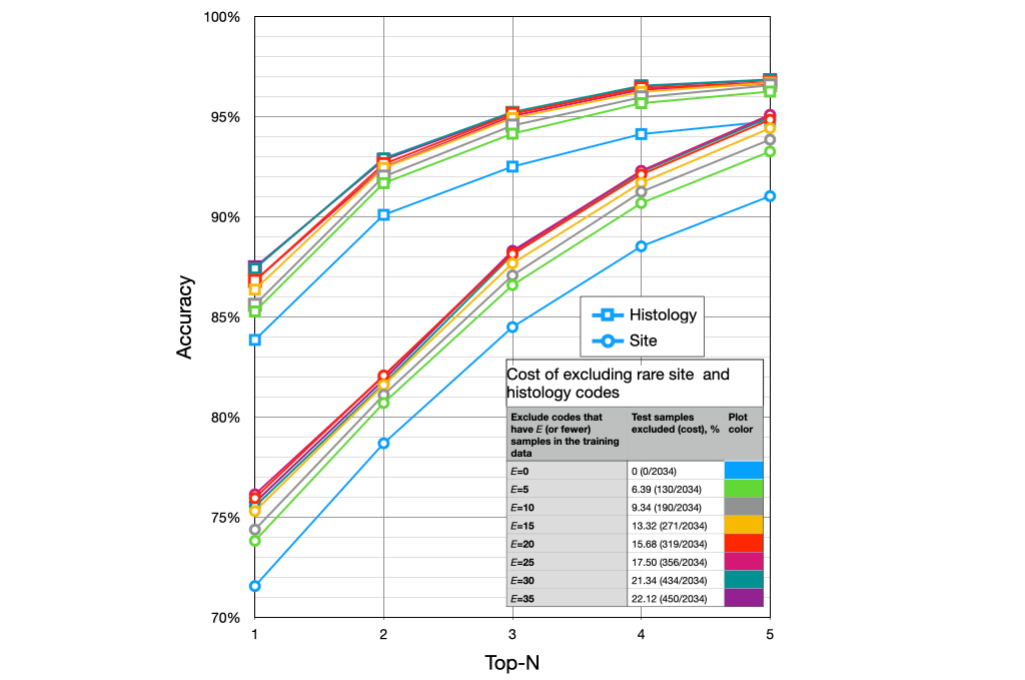
The effect of culling rare tumor sites and histologies on the top N accuracy of predicting fine-grained International Classification of Diseases for Oncology, Third Edition codes.

The top N accuracy of predicting fine-grained site codes ranged from 71.58% (1456/2034; top 1) to 91.05% (1852/2034; top 5), without culling (Figure 5). The accuracy for predicting histology codes ranged from 83.87% (1706/2034; top 1) to 94.79% (1928/2034; top 5). Culling 6.39% (130/2034) of the test samples—those site and histology codes with <5 samples in the training data set—improved accuracy for site code prediction to 73.84% (1406/1904; +2.26%; top 1) and 93.28% (1776/1904; +2.23%; top 5). The same culling improved the accuracy of histology code prediction to 85.29% (1624/1904; +1.42%; top 1) and 96.27% (1833/1904; +1.48%; top 5).

### Group-Level Code Predictions

**Figure 6:**
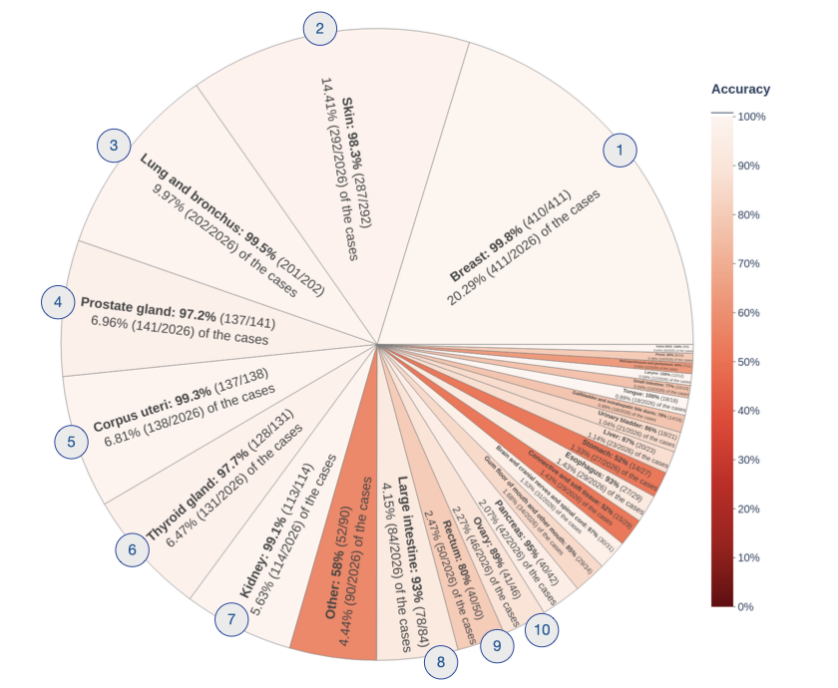
Accuracy of predicting tumor site group codes from unstructured and previously unseen pathology reports on solid tumors, broken down to show performance within each site group. The overall accuracy over all site groups was 93.53% (1895/2026).

We also computed the accuracy of the more coarse–grained group-level code predictions by mapping each top 1 code prediction to its corresponding group in the ICD-O-3 ontology. The accuracy of predicting group-level site codes was 93.53% (1895/2026) overall (Figure 6). The ten most commonly represented sites—(1) breast, (2) skin, (3) lung and bronchus, (4) prostate gland, (5) corpus uteri, (6) thyroid gland, (7) kidney, (8) large intestine, (9) rectum, and (10) ovary—included 79.42% (1609/2026) of the test samples and had an accuracy of 97.7% (1572/1609). Accuracies <80% were observed for connective and soft tissues (15/29, 52%, with 29/2026, 1.43%, of the samples), stomach (14/27, 52%, with 27/2026, 1.33%, of the samples), gallbladder and extrahepatic bile ducts (14/18, 78%, with 18/2026, 0.89%, of the samples), small intestine (10/13, 77%, with 13/2026, 0.64%, of the samples), retroperitoneum and peritoneum (7/11, 64%, with 11/2026, 0.54%, of the samples), and other (52/90, 58%, a collection of 27 sites totaling 90/2026, 4.44%, of the samples).

**Figure 7:**
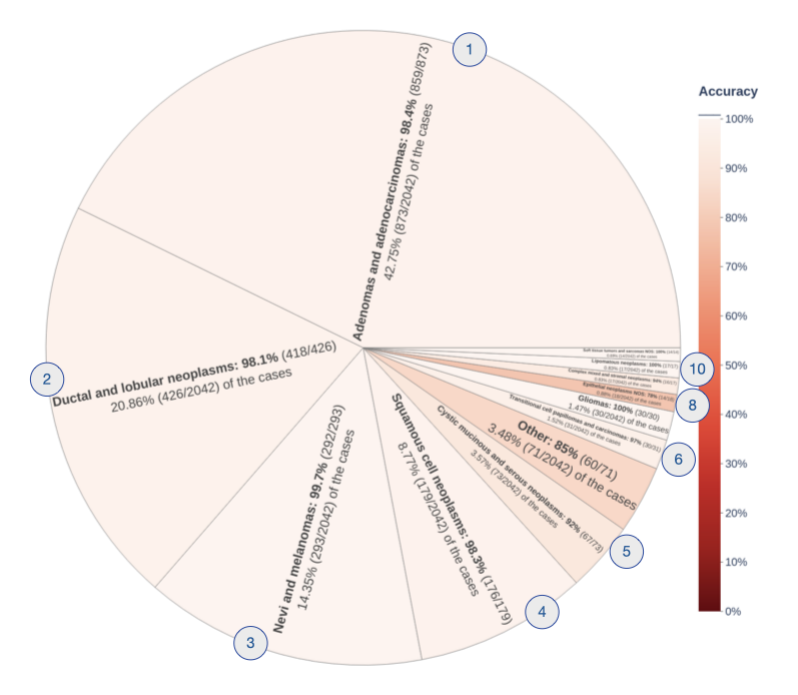
Accuracy of predicting tumor histology group codes from unstructured and previously unseen pathology reports on solid tumors, broken down to show performance within each histology group. The overall accuracy over all histology groups was 97.6% (1993/2042).

The accuracy of predicting group-level histology codes was 97.6% (1993/2026) overall (Figure 7). The ten most commonly represented histologies—(1) adenomas and adenocarcinomas, (2) ductal and lobular neoplasms, (3) nevi and melanomas, (4) squamous cell carcinomas, (5) cystic mucinous and serous neoplasms, (6) transitional cell papillomas and carcinomas, (7) gliomas, (8) epithelial neoplasms, (9) complex mixed and stromal neoplasms, and (10) lipomatous neoplasms—included 95.84% (1957/2042) of the test samples and had an accuracy of 98.06% (1919/1957). An accuracy of <80% was observed for epithelial neoplasms only (14/18, 78%, with 18/2042, 0.88%, of the samples).

## Discussion

### Primary Contributions

This work describes 3 primary contributions. First, we created caBERT: a BERT-based language model for comprehension of cancer pathology reports. We are aware of only 1 other attempt to create a pathology- and oncology-specific BERT language model [45]. That study included 290,438 pathology reports created between 2005 and 2015 from a tertiary teaching hospital in the United States. However, only 8870 of these reports were cases involving cancer. Our study included 275,605 pathology reports from patients with cancer diagnosed or treated at Moffitt. The larger corpus of cancer-specific reports should help our system achieve higher performance levels with cancer-related NLP tasks. However, because we did not have access to the system described in the study by Ma et al [45], a direct comparison with ours was not possible.

Second, we created a new Q&A system to extract tumor site and histology descriptions from free-text pathology reports. This is the first functional Q&A system for extracting information from pathology reports that we are aware of. The Q&A format has 2 important benefits. First, it provides a user-friendly interface to the information extraction system. Second, incorporation of additional questions into our system is straightforward. With appropriate ground truth–labeled training data, this should allow us to extract additional data fields from free-text pathology reports.

Third, we created a new caBERT network, caBERTnet, to predict fine-grained ICD-O-3 site and histology codes using the answers extracted through the initial Q&A component. There has been considerable prior work using NLP methods to predict ICD-O codes from pathology reports [11,12,16,67]. Here, we compare our results to 5 of the most highly cited recent publications in this area.

### Comparisons With Prior Work

Much of the prior work has focused on a single anatomical site or histology. For example, Coden et al [16] described a system to extract information on tumor site, histology, grade, lymph nodes, tumor size, and reporting date from free-text pathology reports of colon cancer. They achieved precision and recall values of between 0.95 and 0.98 for both site and histology ICD-O codes. Their system used a rule-based NLP pipeline, with a large number of controlling parameters that required extensive manual tuning to obtain optimal results. In contrast, BERT-based NLP systems can both discover and tune the steps of a traditional NLP pipeline automatically [68]. This has significant advantages in terms of reduced effort; in addition, it allows these systems to be quickly retuned for data sets from other institutions or different applications through transfer learning [69].

The BERT system from Ma et al [45], mentioned earlier, was used to extract information on 15 *primary cancer sites*, 6 *cancer reasons*, and 3 *metastatic disease* states. In all, 11 of their cancer sites corresponded to ICD-O-3 group-level site classifications (eg, breast, lung, or bronchus). The others were broader groupings (eg, colorectal, upper gastrointestinal, and head and neck). Of their cancer reasons, 4 corresponded to ICD-O-3 group-level histology classifications (eg, melanoma and soft tissue sarcoma). The remaining two cancer reasons were very broad groupings: carcinoma and blastoma. They achieved accuracies on the full test set of 96.7% and 98.5% for cancer site and cancer reason, respectively. However, they did not predict ICD-O-3 group or fine-grained codes.

Nguyen et al [70] developed a system to monitor Health Level Seven International electronic pathology reports from across the state of Queensland in Australia. Their system relied on business rules and symbolic reasoning using Systematized Nomenclature of Medicine codes. They tuned their system using 201 pathology reports and tested it on 220 unseen reports. They extracted 8 different cancer characteristics from these reports. These characteristics included ICD-O-3 site codes (both fine-grained, Cxxx, and group, Cxx) and histological type. Their data set included 66 sites and 94 histologies. They achieved F1 scores of 61.1%, 73.2% and 63.7% on fine-grained site codes, group site codes, and histology codes, respectively.

Alawad et al [67] developed a multistage system of deep convolutional neural networks to extract the primary site, histological grade, and laterality from pathology reports. They achieved an F1 score of 77.5% over 12 ICD-O-3 site codes.

Qiu et al [11] also developed a deep convolutional neural network to extract ICD-O-3 codes from breast and lung cancer pathology reports. Training was based on 942 pathology reports annotated by cancer registry experts. The data set included 7 breast sites and 5 lung sites. Of the 12 sites, 6 had at least 50 samples per code. The remaining 6 sites had 10 to 50 samples each. They evaluated their system using a 10-fold cross-validation. Their overall F1 score for predicting tumor sites across all 12 ICD-O-3 codes was 72.2%.

Our study included a greater diversity of cancer cases than previous studies (214 site codes and 193 histology codes), while obtaining similar or better accuracy scores. Many of the site and histology codes in our training data set included ≤5 samples, whereas prior studies reported ≥10 training samples per code. Culling codes from the test set with ≤5 samples in the training set reduced the size of our test data set by 6.39% (130/2034; 130 pathology reports). However, this increased our top-1 accuracy on the test data to 73.84% (1406/1904; +2.26%) and 85.29% (1624/1904; +1.42%) for site and histology, respectively. Our system also ranks and reports the top 5 predictions for ICD-O-3 site and histology codes. This has useful clinical applications: often there is a degree of uncertainty or *hedging* in pathology reports [16]. Listing the top 5 predicted codes could help to reduce this uncertainty. For example, an artificial intelligence–assisted abstraction system that provides the top 5 predicted ICD-O-3 codes for a particular pathology report (in a pull-down menu, for example) could aid the process of abstraction and enhance the workflow in cancer registries. Our top 5 accuracies for fine-grained codes with ≥5 training samples were 92.95% (1794/1930) and 96.01% (1853/1930) for site and histology, respectively.

### Additional Insights From the Results

Figure 5 shows the top 5 results at various levels of rare-code elimination from the test data set, and it provides 3 additional insights. First, as N increased from 1 to 5, the improvement in accuracy for sites was larger than that for histologies. This suggests that there is more uncertainty predicting site codes than in predicting histology codes. Second, eliminating rare codes, for example, going from E=0 (green lines) to E=5 (blue lines; Figure 5), improved site accuracy more than it improved histology accuracy. This suggests that site prediction was more dependent on sample size. Third, site accuracy failed to improve for E>20. This suggests that 20 samples per code were required to maximize site code prediction accuracy.

The overall accuracy for predicting site group codes was 93.53% (1895/2026) (Figure 6). Nevertheless, several site group codes had accuracies <80%. Here, we will discuss the *Other* group (52/90, 58%, accuracy), along with two of the site group codes with the lowest accuracies: *C49 Connective, Subcutaneous, and Other Soft Tissues* (15/29, 52%, accuracy) and *C16 Stomach* (14/27, 52%, accuracy).

The *Other* site category included 27 group codes. Together, these group codes contained 61 fine-grained codes with at least one sample pathology report each in the training data set, as determined by the MCR. The mean and median number of reports in the training data set for each fine-grained code in the *Other* category were 6.3 (SD 6.3) and 4 (IQR 5), respectively. Consequently, caBERTnet accuracy on these rare sites was likely limited by the availability of training data.

caBERTnet failed to predict the MCR site code for 14 test cases in the group *C49 Connective, Subcutaneous, and Other Soft Tissues*. We manually reviewed 50% (7/14) of these cases, all of which were labeled by the MCR as soft tissue of the limb, shoulder, and hip or pelvis (codes *C491*, *C492*, and *C495*). In 14% (1/7) of these cases, the information required to determine the correct site was not present in the pathology report text. In these situations, CTRs would use additional information in the patient record. However, this information was not available to caBERTnet. In the remaining 86% (6/7) of the cases, the pathology report described characteristics of a lesion that had metastasized from the limb, shoulder, hip, or pelvis to another location. The MCR recorded the originating organ as the tumor site, whereas caBERTnet predicted the metastasis site.

caBERTnet failed to predict the MCR site code for 13 test cases in the group *C16 Stomach*. All these cases were labeled by the MCR as *C160 Cardia, NOS*, and by caBERTnet as lesions of the lower third of the esophagus (*C155*; 12/13, 92%) or as overlapping lesions of the esophagus (*C158*; 1/13, 8%). The MCR labels are due to a rule in the American Joint Committee on Cancer Staging Manual, Eighth Edition [71]. On page 189 in that manual it states as follows:

Cancers involving the Esophagogastric Junction (EGJ) that have their epicenter within the proximal 2 cm of the cardia (Siewert types I/II) are to be staged as esophageal cancers. Cancers whose epicenter is more than 2 cm distal from the EGJ, even if the EGJ is involved, will be staged using the stomach cancer TNM (primary tumor, lymph nodes, and distant metastases) and stage groupings (see Chapter 17).

The pathology reports on these cases did not mention the spatial location of the tumor sample in relation to the EGJ. Consequently, measurement of the tumor location in pretreatment imaging was required to determine the correct tumor site code.

The overall accuracy for predicting histology group codes was 97.7% (Figure 7). Only one group code had an accuracy <80%: *801-804 Epithelial Neoplasms, NOS* (77.8%). caBERTnet failed to predict the MCR histology code for 5 of these cases. In 80% (4/5) of these cases, the pathology report was based on histology at the metastatic site of disease. The MCR coded these as the histology of the originating tumor, whereas caBERTnet predicted the histology at the metastatic site. In 20% (1/5) of the cases, the information required to determine the correct histology code was not present in the pathology report and required the CTR to conduct a review of the patient medical record.

The last case was quite interesting because the pathology report included an initial intraoperative diagnosis that disagreed with the final diagnosis. The former indicated a histological type of *spindle cell carcinoma*. The latter included the following statements: “the differential diagnosis includes sarcomatoid carcinoma and inflammatory myofibroblastic tumor...the histomorphologic and immunoprofile support the diagnosis of sarcomatoid carcinoma.” The MCR coded the histology as *8032/3 spindle cell carcinoma, NOS*, based on the intraoperative statements, whereas caBERTnet predicted *8033/3 pseudosarcomatous carcinoma*. The phrase *sarcomatoid carcinoma* is an alternative form of the ICD-O-3 preferred phrase *pseudosarcomatous carcinoma*. Although caBERTnet’s prediction did not agree with that of the MCR, downstream applications may still value automatic prediction and codification of the final diagnosis.

### Potential Applications

There are multiple potential applications of caBERTnet at Moffitt. For example, there is a delay of several months between initial pathology report dictation and CTR abstraction because the CTRs typically wait for enough time to have elapsed for the first course treatment to have been administered to minimize the number of times they have to review the medical record. caBERTnet can be used to extract information from pathology reports in a timelier way, thus facilitating the use of the data for clinical pathway reporting and screening for clinical trials. Furthermore, CTRs only abstract the subset of pathology reports associated with the cancer diagnosis and first-course treatment. caBERTnet could be used to extract information from pathology reports associated with subsequent biopsies and surgeries that would never be manually curated by the CTRs. To facilitate these use cases, we plan to extract tumor site and histology information close to real time and link these values to other patient data stored in our analytics platform. These data can be incorporated into real-time dashboards and data sets for a wide range of decision support and research applications.

We do not believe that caBERTnet will replace CTRs at cancer clinics. Many complex, difficult, and rare cases require intuition and information outside of the pathology report to determine the correct coding. These cases are beyond the scope of an NLP tool. However, caBERTnet may help simplify and accelerate MCR workflows. For example, caBERTnet could preprocess pathology reports to identify the top 5 site and histology ICD-O-3 codes and their corresponding phrases. The phrases could then be highlighted within the report body. In addition, two pull-down menus could be prepopulated with top 5 code predictions: one for site and the other for histology. The CTR could then quickly choose a code from either pull-down menu. If the correct code was not among the top 5, then the CTR would resort to their current workflow, entering this information by hand.

### Limitations of Approach

Although there are immediate applications of the caBERTnet model within our internal workflows, there are a number of aspects of our modeling approach that limit the application of caBERTnet to other use cases. In this section, we provide an overview of several important limitations that we believe should be considered before caBERTnet implementation.

The most critical limitations correspond to issues related to our data curation and preprocessing approaches. In the curation of our Moffitt fine-tuning data set, we restricted the available reports to only those containing a single primary tumor diagnosis. Although this was partly imposed by the nature of the SQuAD Q&A task (which expects a single answer to each question asked of each input), it nonetheless limits the generalizability of our model to reports containing multiple (or zero) positive diagnoses. We are exploring methods of mitigating these issues within the current setup by adjusting the likelihood thresholds for output predictions that could be used to screen out reports with no diagnosis. Another limitation inherent to a Q&A system is the necessity of knowing the precise span of text corresponding to the answer to each question asked of each report. Owing to the sheer number of reports in our fine-tuning data set, it was infeasible to manually curate answer labels. To circumvent this issue, we chose to create our own automated system to determine the answer text in each report. Any such automated preprocessing necessarily leaves a fingerprint on downstream tasks. The drawbacks of our approach in particular relate to the restriction of answers from a predetermined list of possibilities for each site and histology. Although these phrase sets were diverse (and our approach even allowed for permutations of phrasing within these terms), this process nevertheless limits the allowed terminology and is necessarily incomplete.

In addition, a single pathology report may reference several tissue samples (which can be from related or distant sites from the actual site of diagnosis). Although we limited our automated answer-search preprocessing algorithm to only find terms associated with the known diagnosis label for each report, it is possible that the answer found in the report text by the preprocessing corresponds to a false answer from a different sample in the report; this is particularly relevant for cases with multiple samples from related sites (eg, upper outer quadrant breast vs upper inner quadrant breast). When the 2 samples are in the same site (or histology) group, this issue is avoided by outputting the group code; however, this does not help when the 2 samples belong to different groups altogether.

### Future Directions

We plan to continue development of caBERTnet. Of particular interest is extending the system with additional questions and MCR-derived ground truth labels to train it to extract additional tumor characteristics. These include grade, size, involvement of lymph nodes, primary or metastatic status, presence or absence of molecular markers, and others. caBERTnet could also be customized to extract information on hematological malignancies.

A caBERTnet-assisted MCR abstraction tool could also be used for active [72] or human-in-the-loop [73] learning. Briefly, this approach uses human-labeled data to improve the performance of machine learning algorithms over time. It is particularly useful when the subject matter expert (a CTR in our case) provides labels for cases with low-confidence predictions by the machine learning algorithm. However, it would require careful engineering to avoid common pitfalls and ensure seamless operation [74].

The system’s accuracy on rare sites and histologies could be improved with additional training data. A potential option may be to collaborate with other academic cancer centers; distributing caBERTnet for training on local pathology reports at other sites based on ground truth labeling of training and test data sets from highly standardized registry data or the application of federated multitask learning [75] that distributes copies of a central model to multiple spoke sites for tuning of the central model on local data could allow vast improvements in caBERTnet accuracy. Using these methods, information learned at local sites (eg, model weights) is transmitted back to the central node where the information is combined in a pluralistic way that avoids the need to impose consensus on the data distributions at the spoke sites. This allows for both heterogeneity in local data and broad generalizability of the central model. That said, the most effective way to protect private health information when sharing such models remains an unsolved problem, and these kinds of expansions would depend on the development of validated privacy schemes specific to BERT.

### Conclusions

Our new NLP system, caBERTnet, is built around a network of 3 cooperating BERT instances. On a sequestered test data set, it produced top 5 accuracies of 92.95% (1794/1930) and 96.01% (1853/1930) for fine-grained ICD-O-3 site and histology codes, respectively. This level of accuracy is on par with existing systems in the literature, while also being accurate over a broader range of site and histology groups.

Pathology report abstraction systems such as caBERTnet cannot be expected to achieve performance on par with CTRs who abstract data ultimately incorporated into population-based cancer registries [76], given the vast amount of ancillary data from within the electronic health record that is required for thorough abstraction and the subtle nuances associated with coding guidelines. However, caBERTnet could expedite access to timely pathology data needed for disease surveillance, cohort identification, and clinical trial matching. Furthermore, it can improve existing workflows, serving as a valuable step toward the ultimate goal of a mostly automated abstraction system.

## Appendix

Multimedia Appendix 1 Additional information on the CancerBERT and CancerBERT network model training and performance as well as an elaboration on the acceptable phrases used to curate labels for pathology reports in the question-and-answer stage.

## Abbreviations

BERT: bidirectional encoder representations from transformers
BioASQ: Biomedical Semantic Indexing and Question Answering
caBERTnet: CancerBERT network
CTR: certified tumor registrar
EGJ: esophagogastric junction
EMR: electronic medical record
ICD-O-3: International Classification of Diseases for Oncology, Third Edition
MCR: Moffitt Cancer Registry
MIMIC-III: Medical Information Mart for Intensive Care, version 3
NLP: natural language processing
NOS: not otherwise specified
Q&A: question and answer
SEER: Surveillance, Epidemiology, and End Results
SQuAD: Stanford Question Answering Dataset
