# Supplementary material for: A Question-and-Answer System to Extract Data From Free-Text Oncological Pathology Reports (CancerBERT Network): Development Study
Source: J Med Internet Res. 2022 Mar 23;24(3):e27210. doi: 10.2196/27210 (PMC8987958; doi:10.2196/27210)
Supplement: Multimedia Appendix 1 [file jmir_v24i3e27210_app1.docx]

# Multimedia Appendix 1

Our NLP system was developed using Python 3.6 (Anaconda Inc. Austin TX), and the PyCharm integrated development environment (JetBrains Inc., Prague, Czech Republic). We utilized the PyTorch machine learning framework[[47]](https://paperpile.com/c/SN7aaA/Fwbao) and the HuggingFace Transformer[[48]](https://paperpile.com/c/SN7aaA/ylI8n) library. We employed a DGX-1 Deep Learning System (Nvidia Inc, San Jose CA) equipped with 8 x Tesla V100-SXM2-32GB GPUs. This system ran Ubuntu 18.04 LTS and had the CUDA 10.2 (Nvidia Inc.) acceleration framework installed. Training programs were executed inside Docker containers (version 19.03, Docker Inc., Palo Alto CA)

In total, 275,605 unique pathology reports were available for processing. Each pathology report was stored either as a plain-text file, or as an HL7 message. For the latter, we used the “python-hl7” library to extract OBX segments containing diagnoses and comments into plain text files. Prior to training, all plain text files were concatenated into a single training file. A single carriage return marked the end of each report within the training file. This meant that line-by-line processing, an option during training, would cause each report to be processed separately. Otherwise, all reports would be treated as related paragraphs in a larger contiguous document. Also, all text was converted to lowercase and cleaned in order to remove obvious PHI along with a set of special characters (including asterisks, commas, underscores, dashes, tildes, brackets, parentheses, carets, quotes, pipes, accents). Five percent of the reports were selected at random and moved from the training file into a separate evaluation file. These reports were used to provide an independent estimate of neural network loss during training each time log results were saved (every 1,000 steps). No other preprocessing was performed.

We extended the default vocabulary provided with the ClinicalBERT model to add words that appeared frequently in our sample of pathology reports. Vocabulary expansion has been shown to improve language model performance for domain-specific tasks. We followed the general process described previously for extending the English language BERT vocabulary for nuclear science[[49]](https://paperpile.com/c/SN7aaA/cPEMS). However, for our application we used the open source scispaCy package for biomedical, scientific and clinical text processing[[50]](https://paperpile.com/c/SN7aaA/eRcfO) and its “en_core_sci_md” language model. We used this model to construct a vocabulary of words in the pathology report text file described above. This vocabulary contained a list of all unique words in the file, along with the number of occurrences of each word in the file. Next, the pathology vocabulary was filtered to remove any words that appeared in the ClinicalBERT vocabulary. The filtered pathology vocabulary was then sorted by word count, and the 100 most common words identified. Next, 100 unused entries in the ClinicalBERT vocabulary, each marked with an “[unused]” token, were replaced with these common words.

The experimental parameters used to train caBERT are listed in [**Table S1**](#sta_model_params). Two epochs were used for each of the first two Q&A training stages. However, the final Q&A training stage, on Moffitt data, trained over 3 epochs. This value was derived based on hyperparameter tuning using a separate validation dataset that was not part of the training or test set. We found that moving from 2 to 3 epochs increased accuracy. Moving from 3 to 4 epochs reduced accuracy. Increasing the number of epochs to 5 or 6 reduced accuracy further.

## 1.1 Training the Site and Histology Code Classifiers: Additional Information

CR generated ground-truth phrases were labeled, then concatenated to form a combined phrase. For example, if the CR phrases were “lung lower lobe” and “squamous cell carcinoma” then the combined phrase would be “site: lung lower lobe. histology: squamous cell carcinoma.” Labels and punctuation were included in the combined phrase to enhance performance. The standard BERT tokenizer employed in our experiments adds a classification token to each sentence. The two periods in our combined phrase resulted in two classification tokens, allowing more complex relationships between phrases and codes to be represented. The “site:” and “histology:” labels were included to provide clear delineation of the site and histology sub-phrases, and once tokenized, to provide additional focus points for the transformer attention mechanism [[51,52]](https://paperpile.com/c/SN7aaA/r6heL+kl4eJ). This could help caBERTnet learn to leverage site and histology phrases that contained similar or complementary words. For example, given the combined phrase “site: upper lobe lung. histology: with bronchioloalveolar features.” caBERTnet correctly predicted a histology of “8250/3, lepidic adenocarcinoma” and a site of “C341, upper lobe lung”.

The site classification layer was initiated to have 332 labels or classes, one for each of the 332 possible ICD-O-3 site codes. We used a linear lookup table to map, or enumerate, each site code onto a unique integer between 0 and 331. The histology classification layer initialization was similar, except that it had 1,143 labels, one for each ICD-O-3 histology code.

Next, the combined phrase for each sample was tokenized and stored. During training, the input sequence consisted of the tokenized combined phrase. The training label was the enumeration corresponding to the appropriate ICD-O-3 code - the site enumeration for the site classifier, and the histology enumeration for the histology classifier. Cross entropy was used to calculate the loss for each classifier, as is common for multi-label classification tasks[[53]](https://paperpile.com/c/SN7aaA/4YIDD). Each trained classifier returned logits: 332 for the site classifier, and 1,143 for the histology classifier. The logits were converted to probabilities using a softmax function, then sorted to identify and return the 5 highest probability enumerations for each classifier. These enumerations were converted back into ICD-O-3 codes by inverting the application of the code-to-enumeration lookup table.

## 1.2 Supplemental Figures & Tables

| 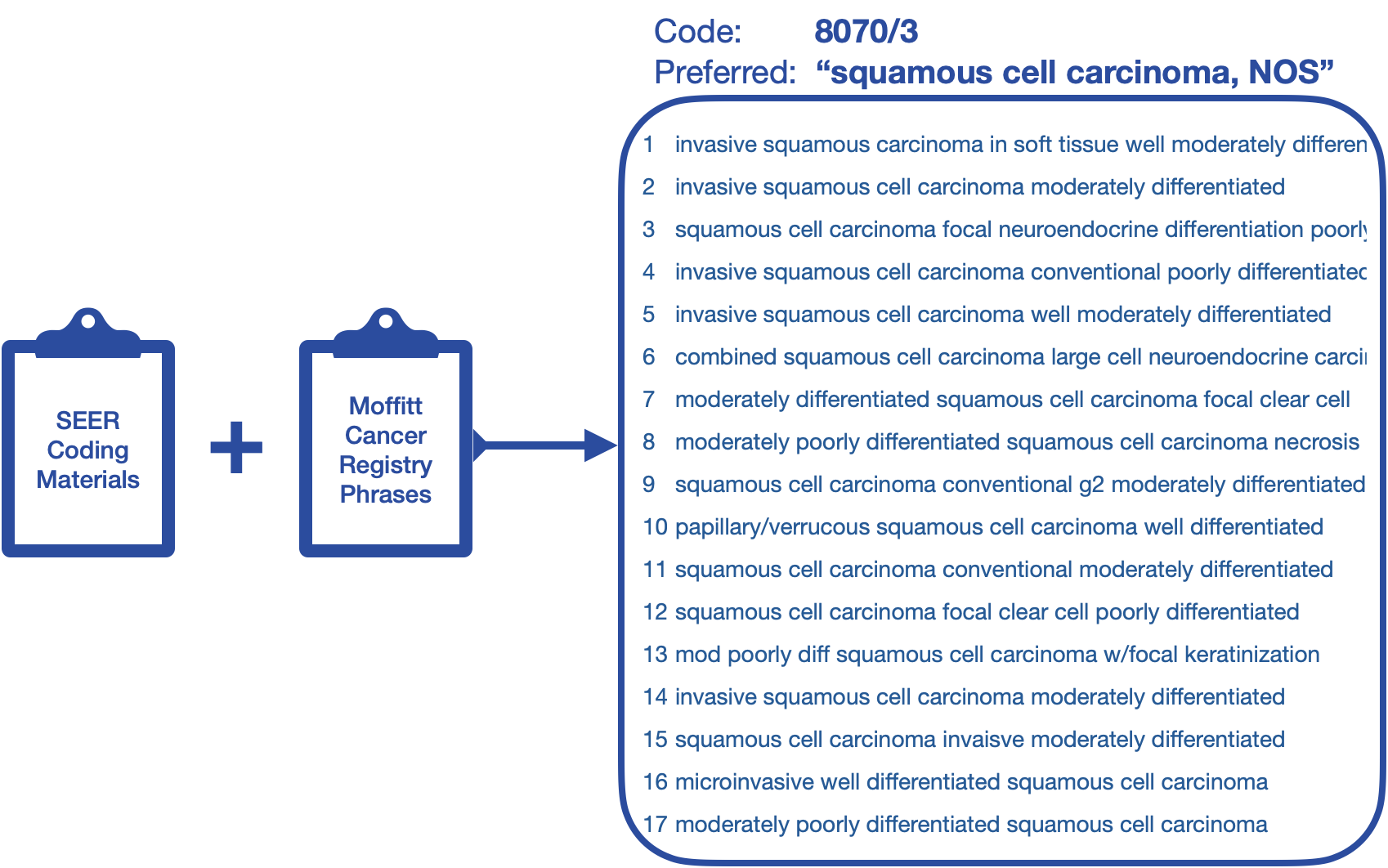 |
| --- |
| [**Figure S1**](#sfigu_phrases) *Construction of the acceptable answer phrase table. Pathology reports used in our training procedures were screened against this table for acceptable “answers” (phrases) corresponding to specific site and histology codes. If a report did not include a match to at least one phrase from this table, it lacked a ground-truth answer, and could not be used for training. This table was constructed starting with the ICD-O-3 and SEER coding materials, which provided the codes, a preferred phrase for each code, and a short list of acceptable alternate phrases. We complemented that with additional phrases from the Moffitt Cancer Registry. In this example, the code “8070/3” corresponds to a preferred phrase of “squamous cell carcinoma, NOS” (No Origin Specified). The phrases in the table labeled 1 through 17 were alternates found in the Cancer Registry that also mapped onto code “8070/3”. Ultimately, code “8070/3” ended up with a total of 161 alternate phrases. The complete table comprised all 332 site codes and 1143 histology codes. After restricting to codes contained in the Moffitt dataset, the final table contained 214 site codes and 193 histology codes, each with a preferred phrase and a list of acceptable alternate phrases.* |

| 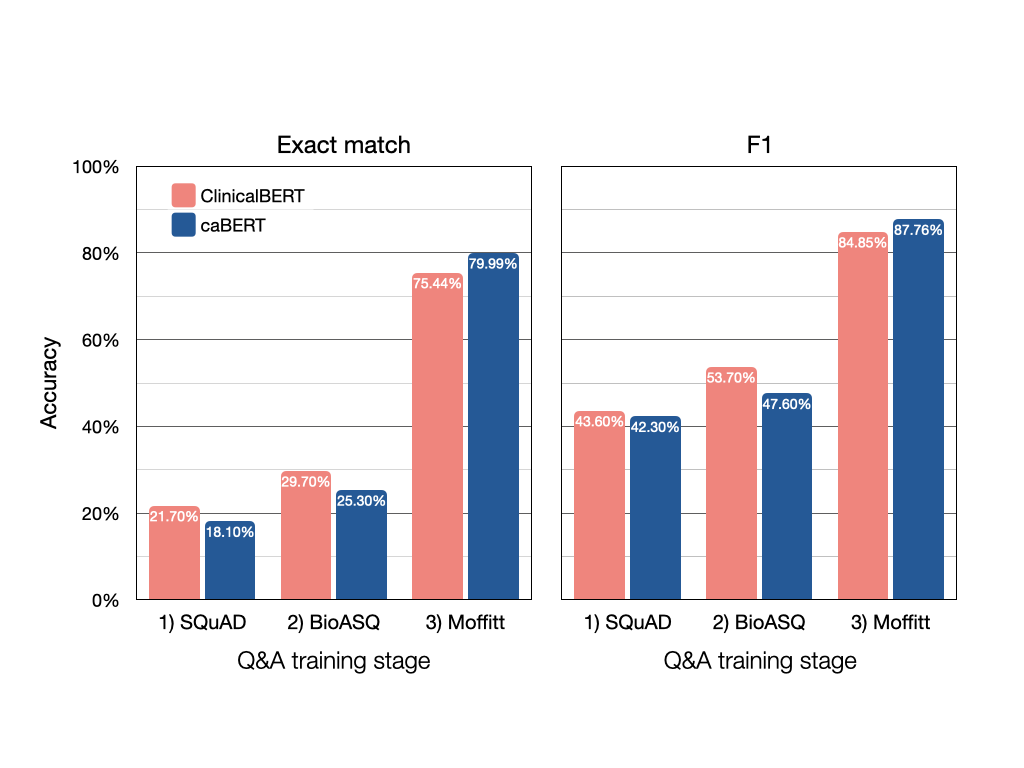 |
| --- |
| **Figure S2** *The impact of training caBERT, a pathology-specific language model, on question-and-answering performance. We trained two Q&A models one with a Q&A layer connected to ClinicalBERT and one with a Q&A layer connected to caBERT. Q&A training proceeded in 3 stages: 1) train it to answer general English language questions with SQuAD v1.1; 2) train it to answer technical biomedical questions with BioASQ 7b; and, 3) train it to answer pathology questions with a local (Moffitt) fine-tuning training dataset derived from our pathology reports, with ground truth provided by our Cancer Registry. At the end of each stage, the system was tested using the Moffitt fine-tuning testing dataset. This contained 2050 sequestered pathology reports, each with ground truth. “Exact Match” refers to a perfect, word-for-word, match to the Cancer Registry phrase. “F1” is the F1 measure of overlap between words in the extracted phrase and the Cancer Registry phrase. This varies from 0 (no words in common) to 1 (all words in common, but not necessarily in the same order), and is expressed here as a percentage.* |

| **Parameter** | **Language Model** | **Question Answering** | **Code Classification** |
| --- | --- | --- | --- |
| Model Type | bert_base_uncased | bert_base_uncased | bert_base_uncased |
| HuggingFace Class | BertForPreTraining | BertForQuestionAnswering | BertForSequenceClassification |
| Train / Validation Split | 95% / 5% | 80% / 20% | 100% / 0% |
| Lower Case | True | True | True |
| Masked Language Model | True | n/a | n/a |
| Initial Learning Rate | 5.0 x 10^-5^ | 3.0 x 10^-5^ | 5.0 x 10^-5^ |
| Learning Rate Decay | Linear | Linear | Linear |
| Weight Decay | 0.1 | 0.1 | 0.1 |
| Warmup Steps | 5,000 | n/a | n/a |
| Training Epochs | n/a | 2 (3 for Moffitt stage) | 10 |
| Training Steps (max steps) | 100,000 | n/a | n/a |
| Logging | Every 1,000 steps | 8 / Epoch | Every 100 steps |
| Saving | Every 5,000 steps | 8 / Epoch | Every 1,000 steps |
| Save Total Limit | 5 | 5 | 5 |
| Number of GPUs | 6 | 8 | 8 |
| Per GPU Train Batch Size | 24 | 3 | 3 |
| Effective Train Batch Size | 144 | 24 | 24 |
| Per GPU Eval. Batch Size | 24 | 40 | 64 |
| Block Size | 512 | 512 | 512 |
| Document Stride | 128 | 128 | 128 |
| Automatic Mixed Precision | True (mode 01) | False | False |
| Line-by-line Processing | True | n/a | n/a |
| [**Table S1**](#stabl_model_params)***:*** *Experimental parameters used to train our caBERT instances. Training and testing leveraged the HuggingFace PyTorch NLP library (v3.03). Training was performed on an Nvidia Inc. DGX-1 with 8 x Tesla V100-SXM2-32GB GPUs. Any parameters not listed used default values.* | | | |

| **Histology** | **Site** |
| --- | --- |
| **805-808 SQUAMOUS CELL NEOPLASMS**   - 8050/0 Papilloma, NOS - 8050/2 Papillary carcinoma in situ - 8050/3 Papillary carcinoma, NOS - 8051/0 Verrucous papilloma - 8051/3 Verrucous carcinoma, NOS   - Condylomatous carcinoma   - Verrucous epidermoid carcinoma   - Verrucous squamous cell carcinoma   - Warty carcinoma   *(Some entries omitted)*   - 8070/2 Squamous cell carcinoma in situ, NOS   - Epidermoid carcinoma in situ, NOS   - Intraepidermal carcinoma, NOS   - Intraepithelial squamous cell carcinoma - 8070/3 Squamous cell carcinoma, NOS   - Epidermoid carcinoma, NOS   - Squamous carcinoma   - Squamous cell epithelioma | **C34 BRONCHUS AND LUNG**   - C340 Main bronchus   - Carina   - Hilus of lung - C341 Upper lobe, lung   - Lingula of lung   - Upper lobe, bronchus - C342 Middle lobe, lung   - Middle lobe, bronchus - C343 Lower lobe, lung   - Lower lobe, bronchus - C348 Overlapping lesion of lung - C349 Lung, NOS   - Bronchus, NOS   - Bronchiole   - Bronchogenic   - Pulmonary, NOS |
| [**Table S2**](#stabl_trees)***:*** *Example of histology and site term hierarchies. Next to each code in the histology and site trees, we list the preferred term as determined by the ICD-O-3.2 table (for histology) and the SEER site-specific coding manual (for site). For each code, we refer to the remaining terms as synonyms. The synonym terms include both the explicit ICD-O-3.2 synonyms as well as terms from the internal Moffitt list of acceptable phrases illustrated in Figure S1. Finally, to each code we also associate histology group and site group labels (located at the base of the tree, in bold), these represent the broad classes of histological morphology and site location.* | |

# 
